# A Systematic Review of Deep Learning and Machine Learning Applications in Longitudinal Multimodal Clinical Data

**DOI:** 10.1007/s41666-026-00248-6

**Published:** 2026-07-10

**Authors:** Jinqian Pan, Tienyu Chang, Mengxian Lyu, Weimin Meng, Qingyu Wang, Yiling Ma, Ziyi Chen, Xiaohan Li, Chengkun Sun, Renjie Liang, Jennifer Fishe, Jie Xu

**Affiliations:** 1 Department of Health Outcomes & Biomedical Informatics, University of Florida, Gainesville, FL USA; 2 Department of BioHealth Informatics, Luddy School of Informatics, Computing, and Engineering, Indiana University, Indianapolis, IN USA; 3 Department of Computer Science, University of Illinois Urbana-Champaign, Urbana, IL USA; 4 Department of Biostatistics, Yale University, New Haven, CT USA; 5 Department of Emergency Medicine, University of Florida College of Medicine – Jacksonville, Jacksonville, FL USA; 6 Center for Data Solutions, University of Florida College of Medicine – Jacksonville, Jacksonville, FL USA

**Keywords:** Artificial intelligence, Machine learning, Deep learning, Multimodal clinical data, Longitudinal data, Systematic review

## Abstract

Artificial intelligence (AI) is increasingly applied in healthcare to support diagnosis and treatment by leveraging longitudinal multimodal data, despite persistent challenges such as missing data and heterogeneity. This systematic review, conducted in accordance with PRISMA guidelines, analyzed deep learning (DL) and machine learning (ML) applications to longitudinal multimodal clinical data published between 2020 and 2024. An initial search identified 1,124 records; a total of 54 items were included (53 studies meeting inclusion criteria and 1 dataset descriptor). Relevant research studies increased substantially over the review period, from 3 studies in 2020 to 21 in 2024. Prediction (n = 23) and classification (n = 19) were the most commonly addressed tasks. Structured electronic health records (EHRs) and medical imaging were the most frequently used data sources, reported in 48 and 33 studies, respectively, and were often used in combination. Regarding missing data, 34 studies reported general handling strategies, and 10 explicitly addressed missing modalities. While methodological variability and performance limitations remain, these findings highlight the growing use of DL and ML to analyze multimodal data, which may inform clinical decision support.

**Supplementary Information:**

The online version contains supplementary material available at https://doi.org/10.1007/s41666-026-00248-6.

## Introduction

The integration of artificial intelligence (AI) into healthcare systems is increasingly influencing conventional approaches to disease diagnosis, treatment planning, and patient management [1, 2]. With advances in machine learning (ML) and deep learning (DL), and the growing availability of large-scale biomedical datasets, AI increasingly supports the development of precision medicine and personalized clinical strategies [3]. Among various AI applications, modeling longitudinal multimodal clinical data—which integrates structured electronic health records (EHRs), medical imaging, genomic and molecular profiles, and clinical narratives—represents a promising yet methodologically challenging area. These data are inherently temporal, heterogeneous, and high-dimensional, posing unique challenges that are well suited for ML- and DL-based solutions [4].

Despite these promising developments, applying ML and DL methods to longitudinal multimodal clinical data remains challenging. First, missing data in real-world clinical datasets can undermine model performance and reproducibility, ranging from partial omissions within modalities to complete modality loss [5]. Second, the heterogeneity and high dimensionality across data types demand advanced frameworks capable of effectively integrating diverse representations and scales [6]. Third, as healthcare AI rapidly evolves, ensuring robustness, interpretability, and cross-domain generalizability across heterogeneous clinical environments remains an ongoing challenge [7]. These challenges are not only data-related but also methodological, requiring innovations in representation learning, temporal modeling, and multimodal fusion.

Given these challenges, several reviews [8–14] have attempted to summarize progress in multimodal or longitudinal learning. However, existing surveys of multimodal machine learning in clinical contexts remain narrow in temporal span, modality coverage, or methodological depth. Many primarily focus on studies published before 2020 [8, 9], offering perspectives that no longer reflect recent methodological advances. Most concentrate on predefined fusion paradigms [10–12]—such as early, joint, and late fusion—or on narrower taxonomies [13] like connection-based, transformation-based, and network-centric strategies, often within limited domains such as neurology or oncology [10]. Consequently, insights from these reviews are seldom generalizable across the broader spectrum of clinical applications. While some reviews discuss longitudinal data, they often emphasize general issues such as data irregularity, heterogeneity, sparsity, and model opacity [10, 15] without examining how these challenges directly inform the design of multimodal fusion frameworks or their cross-domain adaptability. Existing treatments of temporal modeling also tend to focus narrowly on irregular time intervals, missingness, and basic encoding schemes [16], overlooking emerging strategies based on generative modeling, data augmentation, and hybrid temporal architectures. Furthermore, current surveys often restrict their scope to specific tasks (e.g., classification) or methodological families (e.g., self-supervised learning) [14], thereby failing to capture the growing diversity of objectives—predictive, generative, and hybrid—that characterize contemporary multimodal research.

In response to these limitations, this methodological review provides a comprehensive and concept-driven synthesis of recent studies (2020–2024) that apply ML and DL techniques to longitudinal multimodal clinical data, with a particular focus on clinically relevant tasks such as prediction and classification. Unlike earlier reviews that mainly catalog fusion paradigms or summarize progress within individual modalities, our work provides a perspective centered on the interplay among temporal modeling, multimodal fusion, and missing-data handling—three axes that jointly influence the robustness, interpretability, and potential clinical utility of longitudinal AI systems. Within this framework, we systematically examine research trends, task formulations, widely used datasets, fusion architectures, and missingness strategies. By tracing these interdependencies, we outline the current landscape, identify unresolved methodological gaps, and suggest directions to support more rigorous and translational applications of AI in clinical practice.

**Table 1:** Summary of reviewed deep learning and machine learning applications to longitudinal multimodal clinical data

Study	Year	Area	Task	Missing Data	Missing Modality	Fusion Type
Brand et al. [17]	2020	North America	Classification, Prediction			Concat
Gao et al. [18]	2020	North America	Patient-trial matching			Matching
Zhang et al. [19]	2020	North America	Prediction			Concat
Hamdi et al. [20]	2021	Oceania	Classification, Prediction	*\checkmark*		Transformer
Yang et al. [21]	2021	Asia	Prediction	*\checkmark*		Concat
Yang et al. [22]	2021	Asia	Prediction			Concat
Bardak and Tan [23]	2021	Europe	Prediction			Concat
Alsharid et al. [24]	2022	Europe	Video captioning			Concat
Agarwal et al. [25]	2022	North America	Prediction	*\checkmark*		Transformer
Silva and Matos [26]	2022	Europe	Prediction			Concat
Xu et al. [27]	2022	Asia	Prediction	*\checkmark*	*\checkmark*	Concat
Ganjdanesh et al. [28]	2022	North America	Classification		*\checkmark*	Concat
Niu et al. [29]	2022	Asia	Prediction			Concat
Jenefa et al. [30]	2023	Asia	Classification			Concat
Sheetrit et al. [31]	2023	Middle East	Prediction	*\checkmark*		Concat
Budhraja et al. [32]	2023	Oceania	Classification	*\checkmark*		Concat
Mei et al. [33]	2023	North America	Classification, Prediction	*\checkmark*		Concat
Zhang et al. [34]	2023	Asia	Prediction	*\checkmark*		Concat
Tang et al. [35]	2023	North America	Classification	*\checkmark*		Concat
Al-Iedani et al. [36]	2023	Oceania	Prediction			GLMnet
Cai et al. [37]	2023	Asia	Classification			Explainable Boosting Machine
Liu et al. [38]	2023	Oceania	Classification	*\checkmark*	*\checkmark*	Concat
Sun et al. [39]	2023	Asia	Classification			Concat
Bannur et al. [40]	2023	North America	Classification, Generation		*\checkmark*	Concat
Wang et al. [41]	2023	Asia	Classification	*\checkmark*		Concat
Zhang et al. [42]	2023	North America	Classification		*\checkmark*	Concat
Zhu et al. [43]	2023	North America	Generation			Transformer
Rahim et al. [44]	2023	Asia	Classification			Concat
Lu et al. [45]	2023	Asia	Classification			Concat
Nguyen et al. [46]	2023	Europe	Prediction			Concat
Rahim et al. [47]	2023	Asia	Classification	*\checkmark*		Concat
Fang et al. [48]	2023	Asia	Prediction	*\checkmark*	*\checkmark*	Transformer
Du et al. [49]	2024	Asia	Generation			Transformer
Zhong et al. [50]	2024	Asia	Classification			Transformer
Hardan et al. [51]	2024	Middle East	Prediction	*\checkmark*		Transformer
Hl et al. [52]	2024	Asia	Prediction			Concat
Rahim et al. [53]	2024	Asia	Prediction			Soft Voting
Bapat et al. [54]	2024	North America	Prediction			Concat
Ma et al. [55]	2024	Asia	Classification	*\checkmark*		Concat
Gao et al. [56]	2024	Asia	Classification		*\checkmark*	Concat
Zhu et al. [57]	2024	Asia	Classification	*\checkmark*		Concat
Tan et al. [58]	2024	North America	Classification	*\checkmark*		Concat
Aslani et al. [59]	2024	Europe	Classification			Concat
Ding et al. [60]	2024	North America	Prediction			Concat
Rasekh et al. [61]	2024	Europe	Prediction		*\checkmark*	Concat
Chen et al. [62]	2024	North America	Classification, Prediction	*\checkmark*		Concat
Xia et al. [63]	2024	Asia	Prediction	*\checkmark*	*\checkmark*	Concat
An et al. [64]	2024	Asia	Prediction			Concat
Huo et al. [65]	2024	Asia	Recommendation			Concat
Tiwari et al. [66]	2024	Asia	Prediction			Concat
Jeyabalan et al. [67]	2024	Asia	Classification			Concat
Mei et al. [68]	2024	Asia	Generation			Transformer
Wang et al. [69]	2024	North America	Prediction		*\checkmark*	Concat

## Systematic Review Methodology

This systematic review was conducted in accordance with the PRISMA (Preferred Reporting Items for Systematic Reviews and Meta-Analyses) guidelines [70, 71] to support methodological transparency and consistency.

### Operational Definitions

To ensure consistency across study selection, taxonomy development, and reporting, key operational definitions were established prior to screening.

#### Multimodal Data

In this review, multimodal data refers to the integration of fundamentally distinct data types that originate from different measurement domains or acquisition processes. Examples include combinations of structured EHRs, medical imaging (e.g., MRI, CT), clinical notes, physiological signals, or genomic data. Multiple representations of the same modality (e.g., different MRI sequences such as T1 and T2, or multiple derived features from structured EHR data) were classified as *multi-view* data rather than distinct modalities.

#### Longitudinal Modality

A modality was considered longitudinal if it contained temporally ordered observations collected across multiple discrete clinical encounters for the same patient. This includes both intensive longitudinal data (e.g., high-frequency ICU telemetry) and episodic panel data (e.g., irregular EHR visits), as the core challenge of temporal multimodal fusion remains consistent across these sampling frequencies.

Short-duration continuous recordings obtained within a single observation period (e.g., a single ECG waveform or a single telemetry segment) were not classified as longitudinal modalities, as they capture instantaneous physiological states rather than progression across clinically distinct time intervals. Studies were included if at least one modality satisfied this longitudinal criterion.

#### Missing Modality

Missing modality refers to the absence of an entire modality at a given time point or for a given patient, when that modality is otherwise part of the multimodal study design. For example, if a study integrates structured EHR, imaging, and clinical notes, but imaging is unavailable for certain visits or patients, the imaging modality is considered missing. This definition is distinct from missing features within a modality, which refers to incomplete variables within an otherwise present modality.

These operational definitions were applied consistently during screening, data extraction, and methodological categorization.

### Search Strategy

A literature search was performed across five electronic databases—PubMed, Scopus, ACM Digital Library, IEEE Xplore, and Web of Science—to capture relevant studies. The final database search was conducted on January 27, 2025.

The search strategy was implemented using database-specific syntax adapted to each platform, while preserving the same conceptual query structure. Searches were applied to titles and abstracts to ensure reproducibility and consistency; full-text searching was not performed.

The general search logic combined terms related to multimodality, clinical or biomedical context, longitudinal or temporal structure, and machine learning methods. The core conceptual query structure was defined as follows:

(“Multi-modal" OR “Multimodality" OR “Multimodal" OR “Multimodal Fusion" OR “Multimodal Representation Learning") AND (“Biomedical" OR “Medical" OR “Healthcare" OR “Clinical" OR “Biomedicine" OR “Medicine") AND (“Longitudinal" OR “Time series" OR “Temporal" OR “Sequence") AND (“Machine learning" OR “Deep learning")

The complete database-specific search strings, including field restrictions and syntax variations, are provided in Supplementary Table 1.

Following database retrieval, all candidate studies underwent manual screening to confirm eligibility based on predefined inclusion and exclusion criteria, including verification that machine learning or deep learning methods were employed. This process ensured inclusion of studies using specific architectures such as neural networks, including transformer-based models when relevant to multimodal longitudinal modeling.

Only peer-reviewed articles published in English between January 1, 2020, and December 31, 2024, were considered. Studies published prior to 2020 were excluded because advances in computational infrastructure and algorithmic methods in recent years have changed the methodological landscape, making earlier work less directly comparable. Additionally, publicly available multimodal datasets were relatively limited in scale and maturity before 2020, which may reduce their direct applicability to contemporary models.

### Study Selection

Covidence [72] (a web-based systematic review management platform) was used to streamline the study selection workflow. During the title and abstract screening phase, each article was independently assessed by two reviewers using predefined inclusion and exclusion criteria, as summarized below. Articles for which eligibility could not be determined from the title and abstract alone were retained for full-text evaluation. Discrepancies between the two reviewers during both the title/abstract and full-text screening phases were resolved through discussion and consensus. This process ensured consistent interpretation and application of the predefined inclusion and exclusion criteria.

Studies were included if they met all of the following criteria: (1) utilized a clinical dataset; (2) incorporated multimodal data; (3) included at least one modality comprising time-series data; and (4) employed ML or DL techniques.

**Fig. 1:**
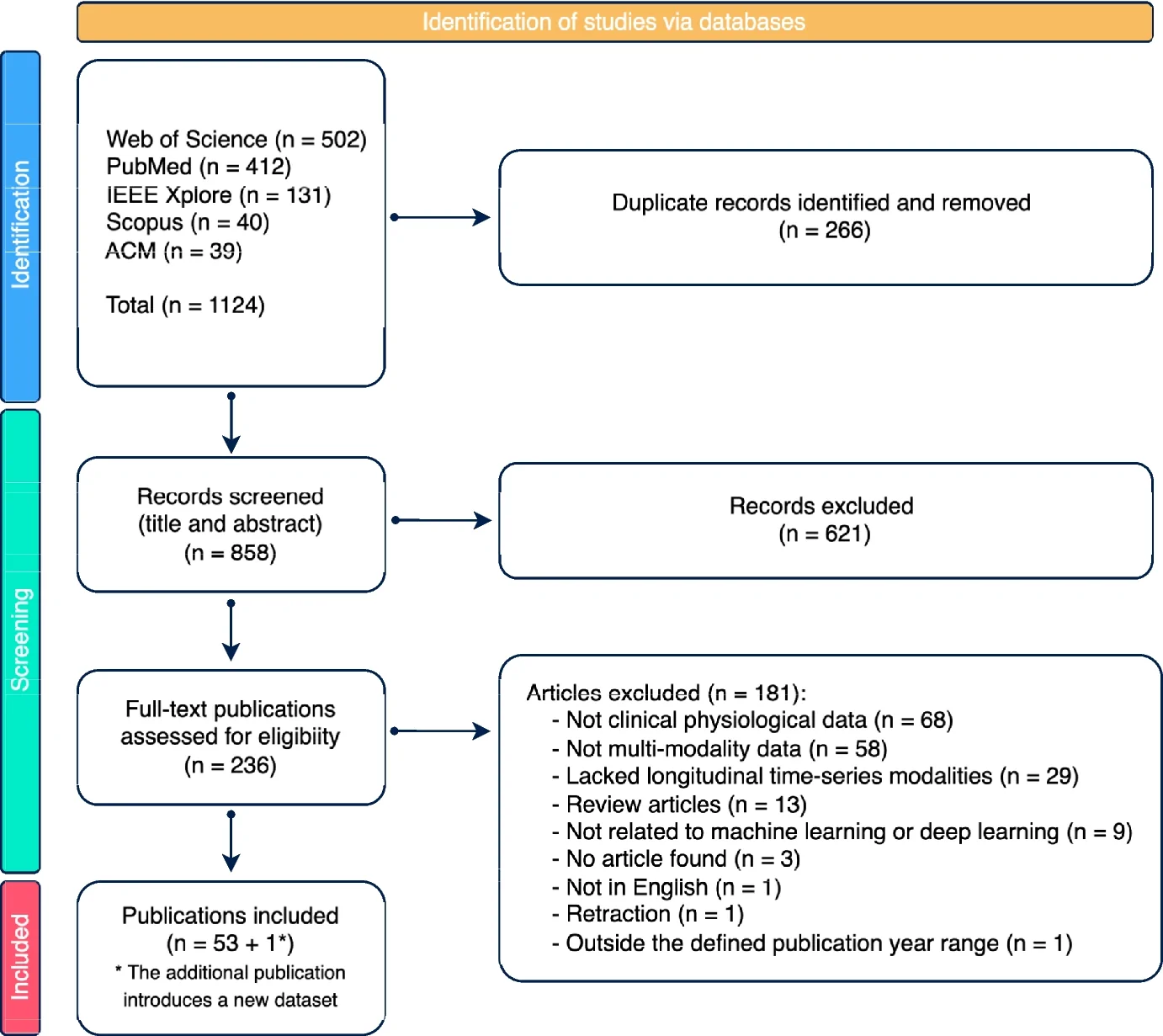
PRISMA flow diagram detailing the identification, screening, eligibility, and inclusion of studies. A total of 1124 records were identified through database searches, with 266 duplicates removed. Of the 858 records screened, 621 were excluded, leading to 236 full-text publications assessed for eligibility. Ultimately, 53 publications were included, plus one additional publication that introduced a new dataset

This review specifically focuses on the multimodal fusion of clinical physiological data, with an emphasis on longitudinal datasets. Accordingly, during the full-text screening phase, studies were excluded if they met any of the following criteria: (1) did not employ clinical physiological data; (2) did not include multimodal input; (3) lacked longitudinal or time-series modalities; (4) were review articles; (5) were not related to ML or DL; (6) were not written in English; or (7) were published outside the defined date range (2020–2024). The PRISMA flow diagram (Fig. 1) illustrates the identification, screening, and inclusion of studies.

### Data Extraction

To facilitate a systematic summary of fusion strategies and data characteristics, we extracted the following key attributes from each included study: (1) country of origin; (2) year of publication; (3) primary clinical or computational task addressed; (4) type of multimodal fusion applied; (5) fusion and embedding architecture; (6) model type or framework employed; (7) availability of publicly released code; (8) use of publicly available datasets; (9) name(s) of the dataset(s); (10) types of modalities included; (11) disease area or clinical condition studied; (12) strategies for handling missing data and missing modalities; and (13) evaluation metrics and validation methods. Extracted variables were subsequently organized according to the three methodological axes (temporal modeling, multimodal fusion, and missing-data handling) defined in Section 2.

## Results

### Descriptive Statistics and Study Selection Overview

Building on the methodology described in Section 2, we summarize here the overall characteristics and distribution of the studies included in this review. The study selection process is summarized in a PRISMA flow diagram (Fig. 1). The initial search across five databases identified 1,124 records. After the removal of duplicates, 858 unique articles remained for title and abstract screening. Based on the predefined inclusion and exclusion criteria, 621 articles were excluded at the title and abstract screening stage because they did not meet one or more of the core eligibility criteria. The most common reasons included studies that did not involve multimodal data, did not include longitudinal or time-series modalities, did not employ machine learning or deep learning methods, or were not conducted in a clinical or healthcare context. The remaining 236 articles proceeded to full-text review for detailed eligibility assessment, as summarized in the PRISMA flow diagram (Fig. 1). As shown in Table 1, following full-text screening, 54 studies met all eligibility criteria and were included in the final analysis, comprising 53 research articles and 1 dataset descriptor.

**Fig. 2:**
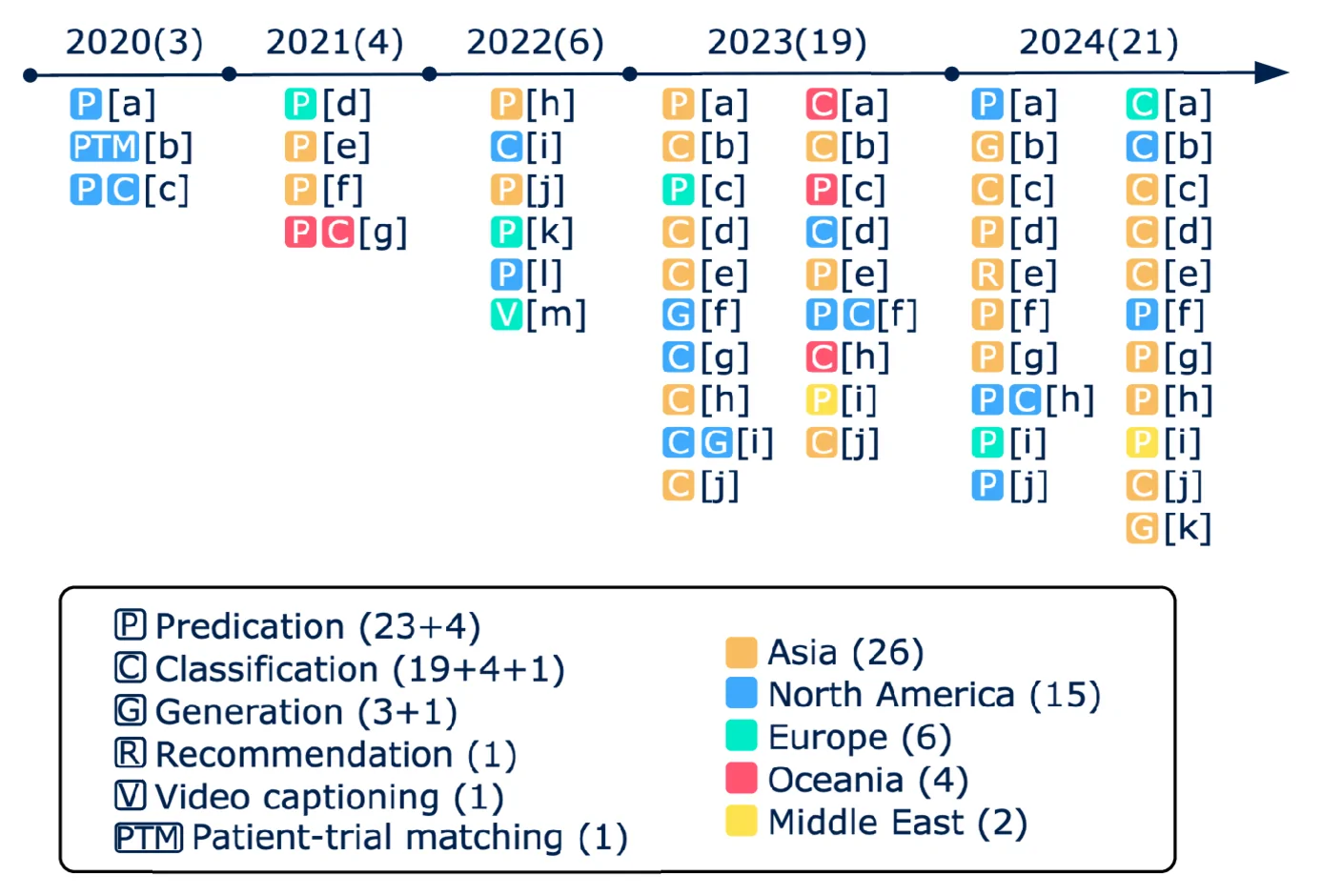
Research publication trends and characteristics (2020-2024). The figure illustrates the annual distribution of research publications from 2020 to 2024, showing 3 papers in 2020, 4 in 2021, 6 in 2022, 19 in 2023, and 21 in 2024. Each publication is categorized by its primary research task(s). The most common tasks include prediction (23 single-task papers), classification (19 single-task papers), and generation (3 single-task papers). Additionally, 4 papers address both prediction and classification, while 1 paper combines classification and generation. Other distinct tasks, each represented by one paper, are Recommendation, Video captioning, and Patient-Trial Matching. The geographical distribution of these publications shows Asia as the dominant region with 26 papers, followed by North America (15 papers), Europe (6 papers), Oceania (4 papers), and the Middle East (2 papers)

Figure 2 summarizes the annual publication trend and regional distribution of the included studies. An analysis of the included studies published between 2020 and 2024 shows an increasing number of research publications, with 3 studies in 2020, 4 in 2021, 6 in 2022, 19 in 2023, and 21 in 2024 (Fig. 2). Each study was categorized according to its primary research objective. Among the most prevalent task types—Prediction (P), Classification (C), and Generation (G)—23 studies focused exclusively on prediction, 19 on classification, and 3 on generation. Additionally, 4 studies addressed both prediction and classification, while 1 study combined classification and generation. For clarity, those overlapping study types were counted in all applicable categories but were not duplicated in the total study count. Other task types, each represented by a single study, included Recommendation (R), Video captioning (V), and Patient-Trial Matching (PTM). Geographically, the majority of studies originated from Asia (n = 26), followed by North America (n = 15) and Europe (n = 6). Oceania (n = 4) and the Middle East (n = 2) contributed smaller but notable shares, reflecting a growing global interest in multimodal learning for longitudinal clinical data.

**Fig. 3:**
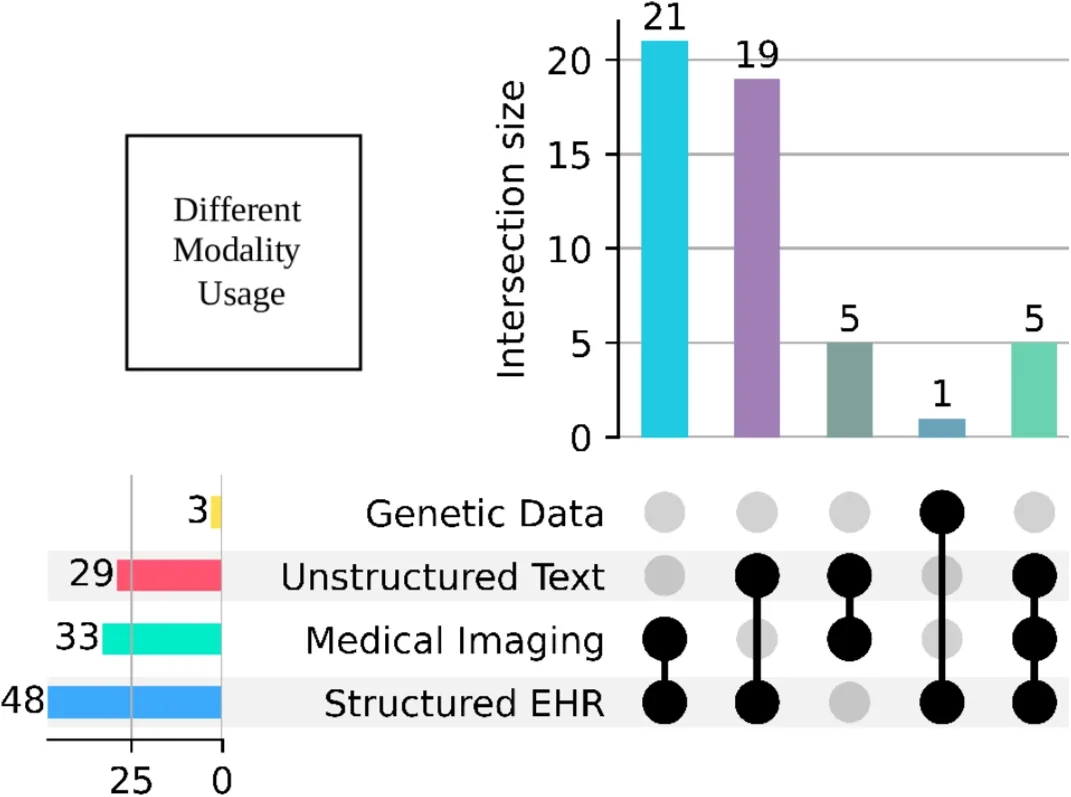
UpSet plot of multimodal data modalities. The left-side horizontal bar chart displays the count of studies utilizing each of the four data modalities. The central matrix illustrates the intersections of these modalities, with filled dots indicating the modalities included in each combination. The top bar chart quantifies the number of studies for each modality combination

To characterize the data landscape of the reviewed studies, we analyzed the frequency and combination patterns of data modalities and their sources. Figure 3 presents an UpSet plot summarizing the intersections of data modalities. The left-side horizontal bar chart displays the overall frequency of the four data modalities identified in this review: Structured EHR (n = 48), Medical Imaging (n = 33), Unstructured Free Text (n = 30), and Genetic Data (n = 3). Only those four data modalities were observed among the included studies. A study was considered multimodal if it incorporated two or more of those modalities.

The central matrix illustrates the specific modality combinations employed in individual studies, while the upper bar chart quantifies the number of studies corresponding to each intersection. The most common combination was Structured EHR and Medical Imaging, reported in 21 studies. This was closely followed by Structured EHR and Unstructured Text, observed in 19 studies. Other notable combinations included Medical Imaging and Unstructured Text (n = 5), and the integration of Structured EHR, Medical Imaging, and Unstructured Text (n = 5). Less frequently observed pairings involved Genetic Data and Structured EHR (n = 2), and Genetic Data and Medical Imaging (n = 1).

**Fig. 4:**
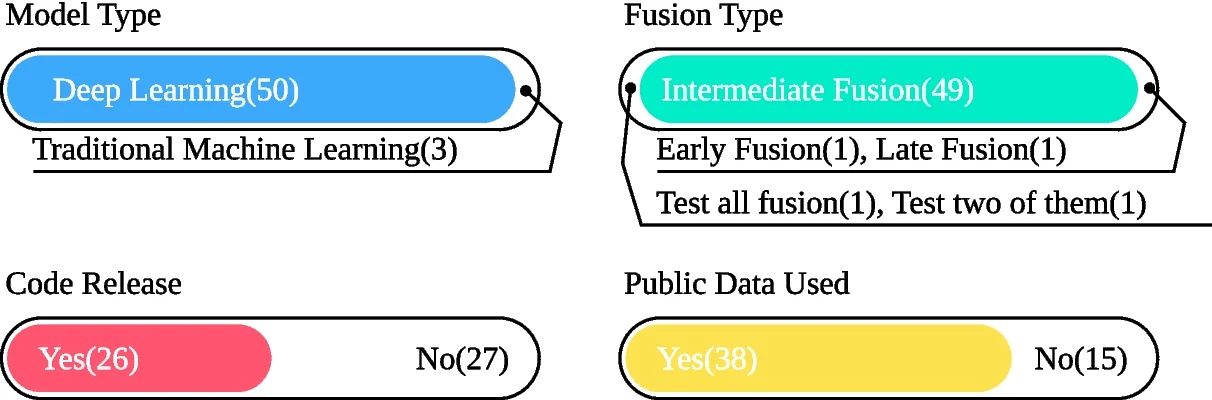
Additional study attributes include Model Type and Fusion Type. Code was publicly released for 26 studies, and public datasets were used in 38 studies

The Fig. 4 provides additional attribute information for the included studies. In terms of model type, DL was the predominant approach, employed in 50 studies, whereas traditional ML methods were used in only 3 studies. Regarding fusion strategies, intermediate fusion was the most commonly adopted, appearing in 49 studies. Fusion stage categories (early, intermediate, and late fusion) were treated as mutually exclusive, and each study was assigned to a single primary fusion stage. In contrast, early fusion, late fusion, test-all fusion, and hybrid approaches combining early and late fusion were each observed in only one study. Overall, the dominance of intermediate (and attention-based) fusion indicates a trend toward architectures that can model cross-modal dependencies in longitudinal settings. The plot also highlights reproducibility and data accessibility: 26 studies provided publicly available code, while 27 did not disclose their code. Similarly, public datasets were utilized in 38 studies, whereas 15 studies relied on privately collected data sources.

**Fig. 5:**
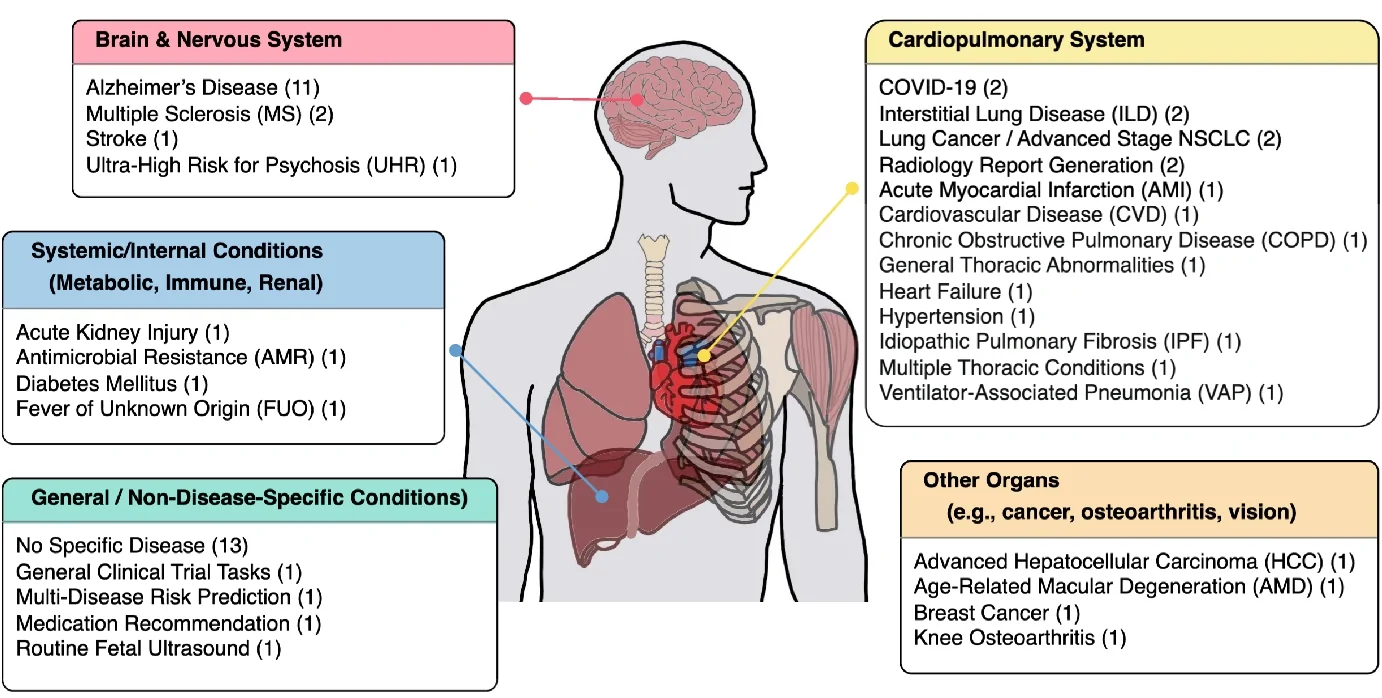
This figure categorizes the diseases studied by body system and displays the corresponding number of studies

The categorization of diseases and associated body systems under investigation, along with the number of corresponding studies, is illustrated in Fig. 5. The most represented category was “No Specific Disease," encompassing 13 studies. That group primarily addressed general clinical or operational outcomes, such as predicting readmission rates, mortality, in-hospital mortality, and length of stay. Alzheimer’s Disease (AD) was the most frequently studied specific condition, featured in 11 studies.

Within the Brain and Nervous System category, in addition to AD, Multiple Sclerosis was investigated in 2 studies, while stroke and high risk for psychosis were each addressed in 1 study. The Systemic/Internal Conditions category, which includes metabolic, immunological, and renal disorders, comprised studies on acute kidney injury, Antimicrobial Resistance, diabetes mellitus, and Fever of Unknown Origin, each appearing in 1 study. In the Cardiopulmonary System, COVID-19, Interstitial Lung Disease, and lung cancer/advanced-stage Non-Small Cell Lung Cancer were each featured in 2 studies. Other conditions in that category—including Acute Myocardial Infarction, Cardiovascular Disease, Chronic Obstructive Pulmonary Disease, heart failure, hypertension, Idiopathic Pulmonary Fibrosis (IPF), Ventilator-Associated Pneumonia, general thoracic abnormalities, and multiple thoracic conditions—were each addressed in a single study. The Other Organs category included studies focused on advanced Hepatocellular Carcinoma, Age-related Macular Degeneration, breast cancer, and knee osteoarthritis, each represented once. In addition to the “No Specific Disease" group, a set of studies addressed broadly defined or non-disease-specific topics, such as general clinical trial tasks, multi-disease risk prediction, medication recommendation, and routine fetal ultrasound; each of these topics appeared in 1 study.

### Data Sources and Modalities

To further contextualize the sources of evidence, we distinguish between public and proprietary datasets and summarize modality usage accordingly. Figure 4 shows that 38 studies utilized publicly available datasets, while 15 relied on self-collected or proprietary data sources. Among the public datasets, the Medical Information Mart for Intensive Care (MIMIC) was the most frequently used, cited in 21 studies, and those studies predominantly addressed topics categorized under “General/Non-Disease-Specific Conditions” in Fig. 5. In contrast, all 11 studies focusing on AD exclusively employed the Alzheimer’s Disease Neuroimaging Initiative (ADNI) dataset. Several other datasets were each referenced by a single study, including the National Alzheimer’s Coordinating Center dataset, Osteoarthritis Initiative dataset, Age-Related Eye Disease Study dataset, ClinicalTrials.gov, proprietary IQVIA claims data, MS-CXR-T, National Lung Screening Trial, SUMMIT Lung Cancer Screening Study, Longitudinal Youth at Risk Study, and the OSIC Pulmonary Fibrosis Progression dataset. Because several works used multiple datasets, the total number of datasets reported exceeds the number of studies. We further discuss the most widely used datasets—MIMIC and ADNI—along with GRAPE [73], a newly published dataset in 2023 identified among the 54 included studies.

Among the 21 studies utilizing MIMIC data, the majority employed MIMIC-III [74], MIMIC-IV [75], or MIMIC-CXR [76] with only a small number using other variations. MIMIC provides rich multimodal clinical data, including structured EHRs, unstructured clinical notes, and medical imaging, supporting a wide range of research tasks from traditional tabular modeling to advanced multimodal learning. In contrast to MIMIC, which provides general-purpose clinical data across a wide spectrum of disease categories, the ADNI is a specialized longitudinal dataset focused exclusively on AD. ADNI provides a comprehensive collection of multimodal data, including structural and functional neuroimaging (e.g., MRI, PET), structured clinical data (e.g., cognitive test scores, clinical assessments), cerebrospinal fluid (CSF) and blood-based biomarkers (e.g., amyloid-*β*, tau), and genomic information (e.g., APOE genotyping). Huang et al. [73] introduced the GRAPE (Glaucoma Real-world Appraisal Progression Ensemble) dataset, a publicly available, longitudinal, multimodal resource specifically designed for glaucoma progression research. For each visit, GRAPE provides a comprehensive set of data modalities, including Visual Field measurements indicative of functional vision loss; color fundus photographs with expert annotations of the optic disc and cup; Optical Coherence Tomography readings; and structured clinical variables such as intraocular pressure, diagnostic labels, and demographic information. In terms of modality classification, GRAPE integrates medical imaging with structured EHR data—including Visual Field measurements, Optical Coherence Tomography-derived Retinal Nerve Fiber Layer thickness, Intraocular pressure, clinical diagnoses, and patient demographics—making it an example of a domain-specific, publicly accessible dataset suitable for longitudinal multimodal analysis.

### Missing Data Strategies

Consistent with the operational definitions established in the methodology, missing modality refers specifically to the absence of an entire modality at a given time point or patient level, rather than missing features within a modality. We next examined how the reviewed studies handled missingness at both the feature and modality levels. We categorize the treatment of missing data in the reviewed studies into two primary areas: (1) handling of missing values within a single modality, and (2) strategies for addressing the absence of entire modalities along a shared temporal axis.

#### Missing Data within a Modality

Among the 53 reviewed studies, the majority (34/53) either removed samples with missing values during preprocessing or did not explicitly report their missing data handling strategies. The remaining 19 studies employed one or more of five main imputation approaches for time-series data: (1) simple statistical methods; (2) FIDDLE (FlexIble Data-Driven pipeLinE); (3) time-based interpolation; (4) model-based imputation; and (5) record-level merging techniques.

(1) Simple statistical methods. Five studies [33, 35, 47, 58, 63] implemented the Last Observation Carried Forward (LOCF) method [77], a simple and widely used approach for handling missing values in longitudinal data. The principle of LOCF is to impute a missing value at time *t* with the most recently observed value at time *t-1*, carrying it forward until a new measurement becomes available. This method is easy to implement, computationally efficient, and preserves the temporal sequence of data while avoiding case deletion. However, it assumes that the underlying value remains constant over time, which may not accurately reflect dynamic clinical changes and can lead to underestimated variability.

(2) FIDDLE [78]. FIDDLE, an open-source preprocessing pipeline used in one study [51], learns a unified latent representation from multimodal time series while accounting for missing values. In this pipeline, missing longitudinal features are imputed using a carry-forward strategy, where each unobserved value is replaced by the most recent available observation to ensure complete inputs for representation learning. Unlike the statistical LOCF method, which assumes that values remain constant until the next observation, FIDDLE applies carry-forward imputation primarily as a pragmatic preprocessing step to facilitate downstream model training.

(3) Time-based interpolation. Six studies [20, 21, 31, 32, 55, 62] adopted time-based linear interpolation, which estimates missing values based on the nearest available data points before and after the gap. This method provides a more flexible approximation of temporal trends compared with LOCF and can better reflect gradual changes over time. Nevertheless, it implicitly assumes that changes between two observations follow a linear trajectory, which may oversimplify complex or non-linear clinical processes.

(4) Model-based imputation. Four studies [25, 27, 38, 41] employed neural architectures that leverage contextual information to impute missing data. Two studies [38, 41] adopted GRU-D [79], a recurrent neural network architecture that introduces decay and masking mechanisms to model temporal dependencies under missingness. Specifically, GRU-D incorporates a binary masking vector to indicate observed variables at each time step and a time interval vector to capture the elapsed time since the last observation, thereby leveraging both the presence and timing of missingness through trainable decay applied to inputs and hidden states. One study [25] adopted a BERT-based masked input strategy, in which subsets of condition, drug, or procedure codes were randomly replaced with a special [MASK] token and the model was trained with categorical cross-entropy loss to predict the masked elements from the remaining patient context. Another [27] utilized an LSTM-based sequence learning module to reconstruct incomplete visits, where missing latent representations or cognitive scores were imputed with predicted values generated from a dense layer conditioned on the previous hidden state, combined with a masking vector to replace only the missing components.

(5) Record-level merging techniques. Another study [57] implemented a record-level merging approach, aggregating multiple shorter records within a fixed time window to reduce sparsity and enhance data completeness. This strategy can be regarded as an implicit form of imputation, as it alleviates missingness by consolidating sparse observations into more continuous and informative representations.

For tabular data, only two explicit imputation strategies were reported. For categorical variables, missing values were assigned to a distinct “unknown” category [33, 35], enabling the model to treat missingness as a potentially informative signal. For continuous variables, one study [20] applied mean imputation, replacing missing values with the variable’s average, a standard method under the assumption of missing-at-random (MAR).

#### Missing Modality

Of the 53 studies reviewed, 10 explicitly addressed the challenge of missing entire modalities at specific time points. Two main strategies were identified: (1) Bypassing missing modalities; and (2) Modality reconstruction.

(1) Bypassing missing modalities. Among these, 8 studies [27, 28, 42, 48, 56, 61, 63, 69] adopted a straightforward strategy: when a modality was absent, the model bypassed the corresponding modality-specific module and generated predictions solely on the available modalities. This approach avoids the need for explicit imputation or reconstruction, which may reduce computational complexity and the risk of introducing noisy surrogate data. However, bypassing comes at the cost of discarding cross-modal information, which may limit the model’s ability to capture complementary signals across modalities and can lead to degraded performance when the missing modality is critical for prediction.

(2) Modality reconstruction. The remaining two studies [38, 40] employed reconstruction techniques, replacing the missing modality with a surrogate representation generated by a pretrained model. These surrogate embeddings were either estimated as the mean of similar samples or inferred conditionally based on the observed modalities. This strategy preserves a unified multimodal representation and allows the model to leverage cross-modal interactions even in the presence of missing data. Nevertheless, its effectiveness depends on the quality of the pretrained model and the assumptions underlying the reconstruction process; poorly estimated surrogates may introduce bias or noise that could affect predictions. Collectively, these findings suggest a trend from ad-hoc imputation toward generative and masking-based approaches that account for uncertainty.

### Fusion Architectures

This section summarizes the principal multimodal fusion strategies implemented across the reviewed literature.

#### Fusion Taxonomy and Counting Logic

Fusion strategies were categorized along two complementary dimensions: fusion stage and fusion mechanism. Fusion stage refers to the architectural level at which modalities are integrated (e.g., early, intermediate, or late fusion), and each study was assigned to a single fusion stage category based on its primary integration strategy. These categories were treated as mutually exclusive.

In contrast, fusion mechanism refers to the specific computational approach used to integrate modality representations (e.g., concatenation-based, attention-based, or interpretable fusion). Because a single model may employ multiple fusion mechanisms within the same architecture (e.g., concatenation followed by attention), mechanism categories were not treated as mutually exclusive. Accordingly, individual studies may be counted in multiple mechanism categories. All reported counts and interpretations reflect this distinction.

Across the reviewed studies, multimodal fusion was achieved through a variety of strategies that combine information from different modalities. We identified five main categories: (1) concatenation-based fusion,; (2) attention-based fusion; (3) decision-level fusion; (4) matching-based fusion; and (5) interpretable fusion.

(1) Concatenation-based fusion. A total of 41 studies first encoded each modality with its own embedding model and then concatenated the resulting representations into a single vector. The concatenated vector was either fed directly into a multilayer perceptron (MLP) at the final stage, or integrated at intermediate layers to allow further joint representation learning before prediction. This approach is simple, architecture-agnostic, and ensures that all modalities contribute to the predictive model.

(2) Attention-based fusion. In contrast, 8 studies [20, 25, 43, 46, 48–50, 68] adopted attention-based architectures, such as Transformers, for multimodal fusion. In these models, modality-specific embeddings are integrated through attention mechanisms that dynamically weight features according to their contextual relevance. This enables the network to selectively emphasize more informative signals while down-weighting less useful ones. However, these models are typically parameter-heavy, and their computational complexity grows rapidly with sequence length and the number of modalities; in clinical settings, where sample sizes are often limited, this can pose challenges for effective training.

(3) Decision-level fusion (soft voting). One study [53] implemented soft voting at the final decision stage over multiple classifiers trained on the same fused multimodal features. After integrating all modalities into a shared representation, parallel models (e.g., LSTM, GRU, 1D-CNN) produced class probabilities via softmax, and the final prediction was obtained by averaging these probabilities. This ensemble strategy has the advantage of combining complementary strengths of different architectures, improving robustness and mitigating the risk of model-specific biases. However, because fusion occurs only at the decision level, it does not explicitly enhance feature representations or capture deeper cross-model interactions.

(4) Matching-based fusion. For the patient–trial matching task [18], a unique fusion strategy was introduced that explicitly aligns patient and trial representations. Specifically, learned eligibility criteria embeddings were used to attend over a memory network and retrieve the most relevant matching memory. The final prediction was then made by combining the EC embeddings with the retrieved memory representation. In addition, the framework optimized the distance between patient and trial embeddings to distinguish inclusion from exclusion criteria, enabling dynamic patient–trial alignment. This approach directly targets the matching problem and is designed to improve interpretability and task-specific performance. However, its high degree of specialization makes it less generalizable to other multimodal clinical prediction tasks.

(5) Interpretable fusion. In addition, two studies using traditional machine learning techniques adopted interpretable fusion strategies. One employed Explainable Boosting Machines (EBM) [37], and the other utilized GLMnet [36], both performing feature-level integration with explicit model structures that facilitate transparency. These approaches provide greater interpretability by allowing direct assessment of feature contributions, which is particularly valuable in clinical decision-making. However, their reliance on linear or additive assumptions may limit representational capacity compared to deep learning–based fusion methods, making them less effective when handling highly complex multimodal interactions. Overall, the predominance of intermediate fusion at the architectural level, together with frequent use of attention-based mechanisms, suggests a trend toward architectures that better capture cross-modal dependencies in longitudinal settings.

### Evaluation Metrics and Reporting Practices

Performance evaluation across longitudinal multimodal studies remains highly heterogeneous. Most studies assess discrimination using the area under the receiver operating characteristic curve (AUROC), F1-score, and Accuracy, among other metrics. Table 2 summarizes the mapping between task categories and the metrics most commonly used in recent ML/DL studies. Prediction and classification tasks predominantly rely on discrimination-based measures such as AUROC, F1-score, and Accuracy [21, 34, 41, 44, 48]; however, most studies do not assess temporal stability, calibration, or predictive uncertainty. Generation tasks—including report generation and video captioning—mainly use linguistic similarity metrics such as BLEU, ROUGE, and METEOR [24, 43, 68], which quantify textual correspondence but overlook factual or clinical accuracy. Recommendation tasks are typically evaluated using ranking-based metrics such as F1-score and AUROC [65], whereas patient–trial matching tasks rely on retrieval accuracy or AUROC to measure alignment between EHRs and eligibility criteria rather than actual patient outcomes [18]. Overall, these evaluation practices emphasize discrimination performance, but often do not report calibration, temporal validation, or external reproducibility. Future research would benefit from standardized evaluation frameworks that incorporate uncertainty estimation, cross-dataset benchmarking, and clinician-informed validation to improve transparency and comparability across multimodal AI systems.

**Table 2:** Mapping of task categories to commonly used evaluation metrics in longitudinal multimodal ML/DL studies

Task Category	Representative Metrics	Comments / Limitations
Prediction	AUROC, F1-score, Precision, Recall, Accuracy	Most studies report discrimination metrics (e.g., AUC, F1) but seldom evaluate temporal stability or probability calibration.
Classification	Accuracy, AUROC, Precision, Recall, F1-score	The most common task; few studies assess predic-tion uncertainty or the reliability of probabilistic outputs.
Generation	BLEU, ROUGE, METEOR	Metrics emphasize linguistic similarity or recon-struction fidelity but offer limited insight into clinical interpretability.
Recommendation	F1-score, AUROC	Evaluated mainly by ranking performance; external or clinician-in-the-loop validation is seldom performed.
Video captioning	BLEU, ROUGE, F1-score	Relies on standard NLP metrics; limited evalua-tion of factual correctness or domain-specific accu-racy.
Patient–Trial Matching	AUROC, Accuracy	Performance is assessed by retrieval success rather than patient-level clinical outcomes.

### Conceptual Framework Derived from the Literature Review for Longitudinal Multimodal Learning

Based on qualitative synthesis of the 53 included studies, we developed a conceptual framework for longitudinal multimodal learning. Longitudinal multimodal learning aims to model dynamic patient trajectories by integrating heterogeneous information collected over time from multiple clinical sources. Across the studies reviewed between 2020 and 2024, three methodological axes consistently emerged as the core dimensions shaping model design and performance: *temporal modeling*, *multimodal fusion*, and *missing-data handling*. These dimensions together influence how effectively an algorithm captures temporal dependencies and cross-modal interactions in real-world clinical data, and may partially account for uncertainty. This framework is grounded in the operational definitions of multimodal data, longitudinal modality, and missing modality described in Section 2, ensuring consistency between study selection, taxonomy development, and conceptual synthesis.

#### Temporal Modeling

Temporal modeling governs how patient histories are represented and how disease progression is captured across visits or events. Early studies relied on recurrent architectures such as LSTM and GRU to aggregate per-visit embeddings derived from structured EHR, imaging, or textual data, enabling straightforward sequence learning of clinical trends [21, 22, 44]. Subsequent work introduced time-aware or decay-based extensions (e.g., GRU-D) to encode irregular sampling intervals and informative missingness [38, 41], improving robustness to uneven observation density. More recent approaches increasingly adopt Transformer-based or attention-driven architectures to capture long-range dependencies and contextual interactions across visits [20, 43, 46, 48, 68]. These models often combine positional or elapsed-time encodings with self-attention, allowing dynamic alignment of asynchronous clinical events. Overall, the temporal axis has evolved from simple recurrent aggregation toward flexible, context-aware mechanisms designed to represent short-term fluctuations and long-term trends.

#### Multimodal Fusion

Multimodal fusion determines how heterogeneous data streams—such as laboratory measurements, diagnostic codes, radiological features, and clinical narratives—are integrated into a unified representation. The majority of studies perform intermediate (representation-level) fusion, concatenating modality-specific embeddings before joint modeling through neural networks or Transformers [33, 34, 41, 45, 63]. While conceptually simple, this strategy has proven effective and remains dominant across tasks and datasets. A growing body of research explores attention-based or cross-modal fusion mechanisms, where the model learns to weight modalities dynamically according to their contextual relevance or reliability [20, 25, 43, 48, 68]. Such designs may improve predictive performance and provide insight into modality contributions over time. Some studies further investigate decision-level or hierarchical fusion, aggregating predictions from separate modality-specific learners or adapting the fusion pathway according to data availability [53]. Collectively, these works reflect a gradual shift from static concatenation toward adaptive, attention-driven fusion architectures aiming to better exploit modality complementarity while maintaining transparency.

#### Missing-Data Handling

Handling missingness remains a defining challenge for longitudinal multimodal data. Missing information may occur at multiple levels—from individual features within a modality to entire modalities or visits—and can introduce substantial bias if ignored. Traditional imputation techniques, such as mean substitution, last-observation-carried-forward, or linear interpolation, are still widely used for simplicity [20, 21, 31, 58]. However, an increasing number of studies integrate missingness directly into the learning process. Time-aware recurrent models treat observation masks and elapsed time as inputs, while generative and self-supervised strategies (e.g., variational autoencoders, masked-reconstruction objectives) infer latent representations for incomplete inputs [25, 27, 38]. At the modality level, two complementary strategies have emerged: *bypassing*, where the model operates on available modalities only [28, 48, 63, 69], and *reconstruction*, where surrogate embeddings are generated from observed data to maintain a consistent multimodal interface [38, 40]. Together, these trends reflect a movement toward missingness-aware architectures that consider absence as potentially informative.

#### Interplay of Axes

Although each methodological axis can be examined independently, effective real-world implementations require their co-optimization. Temporal encoding influences how missingness is represented; missing-data mechanisms affect which modalities can be fused; and fusion strategies determine how temporal alignment unfolds across modalities. For example, attention-based temporal models stabilize fusion under irregular sampling by providing reliability-weighted embeddings [38, 46, 48]. Conversely, adaptive fusion can down-weight unreliable or absent modalities, mitigating error propagation through time [33, 43]. Understanding these interactions may inform the design of systems capable of handling heterogeneous, incomplete, and asynchronous clinical data. In this sense, the temporal–fusion–missingness triad serves as a descriptive taxonomy and provides a framework for thinking about future methodological directions and benchmark design in longitudinal multimodal learning.

## Discussion

This systematic review summarizes findings from 53 studies published between 2020 and 2024 that applied DL and ML techniques to clinical longitudinal multimodal data across a range of healthcare tasks, including prediction and classification. Our findings reveal several key trends in healthcare AI research, including temporal growth in research output, task type distributions, geographic contributions, and prevailing data processing methodologies. Together, these trends provide a general view of the current landscape while also highlighting methodological and practical challenges in the development and deployment of multimodal AI systems in clinical settings. Collectively, the reviewed studies suggest an increasing interest in moving from static, cross-sectional analyses to more longitudinal frameworks that model patient trajectories and disease progression. Compared with previous reviews on multimodal learning in healthcare [8, 11], this study specifically focuses on temporal modeling and missingness-aware fusion, emphasizing challenges that arise when modalities are asynchronous or intermittently missing.

The field of healthcare AI has grown notably over the past several years. As illustrated in Fig. 2, the number of relevant publications increased from 3 in 2020 to 20 in 2023 and 21 in 2024. This growth trajectory reflects the convergence of several enabling factors, including the maturation of AI technologies, the increasing availability of healthcare data, and a rising demand for intelligent clinical decision support systems. In terms of research focus, prediction and classification tasks dominate, appearing in 24 and 19 studies, respectively. This emphasis highlights AI’s emerging role in supporting diagnostic and prognostic workflows. The emergence of generative modeling (n = 3) suggests growing interest in data synthesis and simulation for novel medical applications. Moreover, the rise of multitask studies and the inclusion of diverse tasks—such as recommendation, video captioning, and patient–trial matching—reflect an expanding range of use cases (including healthcare / research operations) and increasing task complexity in the healthcare AI landscape.

With respect to data practices, public datasets were used more frequently than private sources, with 38 studies relying on publicly available data and 15 utilizing proprietary or self-collected datasets (Fig. 4). Among public datasets, MIMIC was the most widely adopted, cited in 22 studies—primarily those investigating general or non-disease-specific conditions. All 11 AD studies exclusively used the ADNI dataset, highlighting a high degree of data centralization in the AD research community. These frequently used datasets appear to function as common reference resources within their respective subdomains, supporting reproducibility and standardization. However, beyond these widely used datasets, the majority of remaining sources were referenced by only a single study, indicating substantial dataset fragmentation. That, along with continued reliance on private datasets, poses barriers to reproducibility, benchmarking, model generalizability, and accordingly, ultimate implementation of healthcare AI applications in the clinical setting. Furthermore, the inherent heterogeneity and high dimensionality of multimodal clinical data demand advanced models capable of effectively integrating diverse data types. As the field continues to evolve, unresolved issues related to model robustness, interpretability, and generalizability in real-world clinical settings remain critical challenges.

### Interpretability and Clinical Translation

Interpretability and clinical integration remain limited across the longitudinal multimodal ML/DL studies we reviewed. A small subset of works incorporated explicit visualization or attribution. For instance, attention or saliency maps were used to highlight disease-relevant regions or modality contributions in AD and cancer studies [44, 47, 56], and note-based models visualized section/word importance for ICU outcomes on MIMIC [22]. In interstitial lung disease, attention-based fusion further illustrated prognostic CT regions [33, 50]. Despite these advances, systematic assessment of cross-modal contributions, counterfactual probing, or concept-bottleneck analyses remains rare, leaving most models functionally opaque to clinicians.

External and temporal validation were reported less frequently. A few studies evaluated generalizability across sites or time (e.g., ILD diagnosis/prognosis and multimodal readmission prediction) [33, 35], but most relied on single-site retrospective cohorts. Calibration and reliability metrics (e.g., Expected Calibration Error, Brier score, decision curve analysis) were reported infrequently, reflecting an overemphasis on discrimination metrics such as AUC or F1 without evaluating reliability or uncertainty.

From a translational perspective, clinician-in-the-loop evaluation and workflow studies were seldom described. Even in generation settings that mimic reporting workflows—such as pre-filling radiology reports or multimodal report generation—iterative expert feedback and failure analysis were typically absent [43, 68]. Addressing this gap may benefit from approaches that incorporate human–AI co-evaluation, decision-curve assessment, and alignment of model rationales with clinical reasoning.

Finally, transparency and governance remain areas needing further attention. Across our corpus, code release and detailed reporting varied widely; descriptions of missing-data handling, temporal window definitions, and fusion design were often incomplete. Adopting checklist-style reporting that covers dataset accessibility, missingness strategies, calibration assessment, temporal definitions, and code availability will improve reproducibility and accelerate the translation of multimodal AI from promising prototypes to clinically actionable systems.

### Challenges and Future Directions

Before discussing methodological challenges identified in the reviewed literature, several limitations of this review itself should be acknowledged. Although study screening was conducted independently by two reviewers with discrepancies resolved through discussion and consensus, formal inter-rater agreement statistics (e.g., Cohen’s kappa) were not calculated. In addition, a formal study quality or risk-of-bias assessment was not performed. The primary goal of this review was to synthesize methodological developments in multimodal longitudinal machine learning rather than to evaluate clinical effectiveness across studies. Nevertheless, the absence of structured quality assessment and quantitative inter-rater agreement measures may limit the transparency and methodological rigor of the review.

Across the reviewed literature, missing data emerged as a common challenge. A large proportion of studies either excluded incomplete records or offered limited detail on how missingness was addressed [20, 21, 27, 42, 48]. When strategies were described, they often relied on straightforward statistical techniques such as LOCF or interpolation [20, 33, 35, 63], while more advanced or model-based methods were less frequently applied [25, 38, 41]. In multimodal settings, absent modalities were typically ignored rather than reconstructed [28, 42, 61, 63]. Future work may benefit from approaches that accommodate partially observed data, including multi-view learning frameworks and modular fusion strategies. Comparative assessments of missing data strategies across diverse datasets may also help clarify their relative strengths in real-world clinical contexts.

Dataset fragmentation was another notable issue. While widely used public datasets such as MIMIC and ADNI provided a foundation for many studies [22, 27, 33, 35, 42, 44, 48, 53], most other datasets appeared only once in the corpus [18, 24, 30, 37, 50, 68], and a substantial number were institution-specific or private. This fragmentation constrains reproducibility and complicates benchmarking efforts. Moving forward, greater collaboration to develop interoperable, large-scale repositories—supported by privacy-preserving techniques such as federated learning [80]—may facilitate broader data sharing and potentially improve generalizability.

Another persistent limitation is the lack of standardized evaluation protocols and transparent benchmarking across studies. Considerable heterogeneity exists in the choice of metrics, validation schemes, and temporal definitions, which hinders meaningful comparison of model performance and synthesis of quantitative evidence. To advance methodological rigor and reproducibility, future research should prioritize the establishment of harmonized evaluation frameworks and publicly accessible benchmarks tailored to longitudinal multimodal learning. Such initiatives could support more consistent cross-study comparison and promote transparency, contributing to progress toward clinically applicable and generalizable AI systems.

Concerns regarding model robustness and interpretability were also evident. Deep learning methods dominated, yet many models functioned as black boxes with limited transparency. Few studies incorporated external validation or clinician-in-the-loop evaluation, raising questions about real-world applicability [33, 35, 44, 47, 50, 56]. Advancing the field may involve increased attention to interpretable fusion methods, such as attention-based visualization or counterfactual reasoning, as well as systematic validation across institutions. Research into lightweight architectures and knowledge distillation may further support deployment in resource-constrained clinical environments.

Looking forward, several directions merit consideration. First, integrating multimodal and longitudinal learning with foundation models (e.g., large language models or multimodal transformers) could unify representation learning across structured, textual, and imaging modalities. Second, embedding uncertainty quantification and causal inference into multimodal fusion frameworks will improve model trustworthiness and clinical interpretability. Third, collaboration among clinicians, data scientists, and policy makers is essential for bridging algorithmic innovation with regulatory-grade evaluation and deployment pipelines. Ultimately, advancing these areas may help connect methodological innovation with clinical translation, supporting progress toward more trustworthy, generalizable, and equity-aware AI systems for longitudinal healthcare applications.

## Conclusion

This systematic review provides a comprehensive synthesis of 53 studies published between 2020 and 2024 that employed DL and ML algorithms to process clinical longitudinal multimodal data for tasks such as prediction and classification. Our analysis reflects recent increases in publication activity within the healthcare AI landscape, with research output increasing markedly over the past four years—suggesting continued interest and exploration in this area. The reviewed studies indicate increasing task diversity and participation from multiple regions, with notable representation from Asia. Despite this progress, persistent challenges in data practices remain. These include the fragmented nature of public datasets, continued reliance on private data sources, and insufficient transparency regarding missing data handling strategies. Collectively, these limitations may constrain reproducibility, interpretability, and model generalizability in real-world clinical settings.

In light of these challenges, and considering the inherent limitations of this review (e.g., publication bias and variability in reporting quality), future research may benefit from efforts to develop high-quality, standardized public datasets; improve interpretability and robustness of AI models; increase transparency regarding data practices and missing data handling; and strengthen interdisciplinary collaboration among clinicians, data scientists, and policy experts. Establishing open benchmarks and harmonized evaluation protocols could help enhance methodological rigor and support cross-study comparability. Ultimately, such collective efforts may facilitate progress toward the safe, effective, and equitable integration of AI into clinical practice and support more accurate, personalized, and data-driven healthcare delivery.

## Supplementary Information

Below is the link to the electronic supplementary material.Supplementary file 1 (pdf 64 KB)

## Data Availability

No datasets were generated or analysed during the current study.
