# Supplementary material for: A Systematic Review of Deep Learning and Machine Learning Applications in Longitudinal Multimodal Clinical Data
Source: J Healthc Inform Res. 2026 Jul 10;10(3):627–53. doi: 10.1007/s41666-026-00248-6 (PMC13522311; doi:10.1007/s41666-026-00248-6)
Supplement: Supplementary file 1 [file 41666_2026_248_MOESM1_ESM.pdf]

Supplementary Table 1 Full database-specific search strategies.

Database	Search Fields	Search String
PubMed	Title/Abstract	((”multimodality”[Title/Abstract] OR ”multimodal deep learning”[Title/Abstract] OR ”multimodal fusion”[Title/Abstract] OR ”multimodal representation learning”[Title/Abstract]) AND (”biomedical”[Title/Abstract] OR ”medical”[Title/Abstract] OR ”healthcare”[Title/Abstract] OR ”clinical”[Title/Abstract] OR ”biomedicine”[Title/Abstract] OR ”medicine”[Title/Abstract]) AND (”longitudinal”[Title/Abstract] OR ”time series”[Title/Abstract] OR ”temporal”[Title/Abstract])) AND (english[Filter]) AND (2020:2024[pdat])
ACM Digital Library	Abstract	[[Abstract: ”multimodality”] OR [Abstract: ”multimodal deep learning”] OR [Abstract: ”multimodal fusion”] OR [Abstract: ”multimodal representation learning”]] AND [[Abstract: ”biomedical”] OR [Abstract: ”medical”] OR [Abstract: ”healthcare”] OR [Abstract: ”clinical”] OR [Abstract: ”biomedicine”] OR [Abstract: ”medicine”]] AND [[Abstract: ”longitudinal”] OR [Abstract: ”time series”] OR [Abstract: ”temporal”]] AND [E-Publication Date: (01/01/2020 TO 12/31/2024)]
Scopus	Title, Abstract, Keywords	TITLE-ABS-KEY(”multimodality” OR ”multimodal deep learning” OR ”multimodal fusion” OR ”multimodal representation learning”) AND TITLE-ABS-KEY(”biomedical” OR ”medical” OR ”healthcare” OR ”clinical” OR ”biomedicine” OR ”medicine”) AND TITLE-ABS-KEY(”longitudinal” OR ”time series” OR ”temporal”) AND PUBYEAR >= 2020 AND PUBYEAR <= 2024
IEEE Xplore	Abstract	(”Abstract”:”Multimodality” OR ”Abstract”:”Multimodal Deep Learning” OR ”Abstract”:”Multimodal Fusion” OR ”Abstract”:”Multimodal Representation Learning”) AND (”Abstract”:”Biomedical” OR ”Abstract”:”Medical” OR ”Abstract”:”Healthcare” OR ”Abstract”:”Clinical” OR ”Abstract”:”Biomedicine” OR ”Abstract”:”Medicine”) AND (”Abstract”:”longitudinal” OR ”Abstract”:”time series” OR ”Abstract”:”temporal”) Filters applied: Publication Years = 2020–2024; Content Type = Journals and Conferences
Web of Science	Abstract	AB=(”Multimodality” OR ”Multimodal Deep Learning” OR ”Multimodal Fusion” OR ”Multimodal Representation Learning”) AND AB=(”Biomedical” OR ”Medical” OR ”Healthcare” OR ”Clinical” OR ”Biomedicine” OR ”Medicine”) AND AB=(”longitudinal” OR ”time series” OR ”temporal”) AND Index Date=(2020-01-01 TO 2024-12-31)
